# Tobacco-free policies at worksites in Kansas

**DOI:** 10.1186/s12889-017-4277-9

**Published:** 2017-06-12

**Authors:** Elizabeth Ablah, Frank Dong, Kurt Konda

**Affiliations:** 1 University of Kansas School of Medicine-Wichita, 1010 North Kansas, Wichita, KS 67214 USA; 2 Western University of Health Sciences, 309 E 2nd Street, Pomona, CA 91766 USA

**Keywords:** Tobacco-free, Policy, Environment, Urban, Rural

## Abstract

**Background:**

This study sought to examine the relationship between tobacco-free policies at worksites to worksite demographics such as company size and geographic location.

**Methods:**

Worksites participating in a worksite wellness workshop were asked to complete a worksite wellness instrument, which provided an assessment of their wellness practices already in place in the worksite, including the degree to which tobacco-free policies were in place at the worksite.

**Results:**

At a bivariate level, those more likely to have tobacco-free policies included: urban employers (76.8% versus 50% rural employers, *p* = 0.0001); large employers (> = 250 employees) (74.3% versus 43.1% small employers (<50 employees), *p* = 0.0003); and schools (69.4%) and hospitals (61.5%) (versus 35.5%, agricultural/ manufacturing employers, *p* = 0.0125). At the multivariate level, rural employers (AOR = 0.47, 95% CI 0.23, 0.95) and small employers (AOR = 0.34, 95% CI 0.16, 0.71) had decreased odds, compared to their urban and large employer counterparts, of having tobacco-free policies.

**Conclusions:**

Rural and smaller employers are less likely to have tobacco-free policies than their urban and large counterparts.

**Electronic supplementary material:**

The online version of this article (doi:10.1186/s12889-017-4277-9) contains supplementary material, which is available to authorized users.

## Background

## Tobacco usage and costs

In 2010, 20.2% of adults in the United States were ‘current’ smokers [1] and 25.2% used any tobacco product, such as smokeless tobacco, water pipes, and cigarillos [2]. Tobacco use continues to be the leading cause of preventable death among adults in the United States. The annual average of tobacco-related premature deaths from 2000 to 2004 was 443,000, approximately 18% of all deaths at that time [3, 4]. In the United States, 19.0% of 141 million working adults are current smokers [5].

Beyond the human costs, tobacco use is also costly in terms of healthcare. Healthcare expenditures in the United States topped $1 trillion dollars in 2006 [6], with employers footing approximately one-third of the bill for healthcare expenditures in the United States [7]. More than half of all US workers (55%) received healthcare coverage through their employers in 2011, and 68% of workers either received their healthcare coverage through their employer (or via someone else’s employer) [8]. Because such a large proportion of Americans are covered with employer-based healthcare coverage, worksites have a vested interest in controlling healthcare costs associated with tobacco use in their worksites.

The costs associated with tobacco use are substantial, as smokers cost employers an average of 12 times more than non-smokers [9]. The United States’ direct medical costs and lost productivity attributable to adults smoking exceed $300 billion are $170 billion annually [10]. The annual cost to an employer in the United States is approximately $6000 per tobacco user [11–13]. Smokers make an average of six more visits per year to healthcare facilities, and the dependents of smokers make four more visits per year to healthcare facilities than non-smokers and their covered dependents [14].

## Tobacco-free policies

To curtail workplace smoking and tobacco use among employees, worksites have turned to a range of strategies, from moving designated smoking areas to partial or complete tobacco bans on company property, and even prohibitions on tobacco use at any time, on or off company grounds [10, 15, 16]. Employee tobacco use has been identified as one of the 10 most impactful modifiable risk factors when predicting employers’ healthcare costs [17]. An estimated annual savings of more than $1000 in medical expenses for non-smokers and more than $2000 for smokers can be realized through these types of policies [18].

In addition to the range of tobacco-free policies that a workplace can implement, there are local, state, and national level laws that can support tobacco cessation in the workplace. In locales with workplace smoking bans, employees experience a range of improved health outcomes, including reduced myocardial infarctions [19], improved respiratory functions [20] and sudden cardiac death [21]. Broad public smoking bans can have many more far-reaching impacts, including reduced incidences of asthmatic symptoms in children [22], pre-term births [23], and hospitalization for angina, stroke, and asthma [24, 25].

A tobacco-free workplace can engender cessation attempts among workers [26–29]. Workplaces with tobacco-free policies have employees who are 1.9 to 2.3 times more likely to quit tobacco than employees at worksites in which tobacco is permitted [27]. Predictive modeling of an array of potential anti-tobacco policies and actions indicates that legislatively-mandated total worksite tobacco bans, such as state-level clean air laws prohibiting smoking in any workplace, are among the most efficacious actions that can be taken to reduce smoking prevalence in individuals [30]. Additional factors that can positively influence a worksite’s chances of a successful tobacco ban include proper framing of how bans are communicated [31], the availability of additional outside resources to support the bans [32], and visible upper management support [33].

## Demographics of tobacco users

Rural Americans are more likely than urban Americans to: have ever smoked (48% vs. 41%), currently smoke (22% vs 18%), be lifetime smokeless tobacco users (22% vs 14%), be current smokeless tobacco users (6% vs 2%), or have smoking permitted at least sometimes in their work areas (19% vs 15%) [34]. Males and those who work for a small employer are also at greater risk of using tobacco [35, 36]. In fact, worksites with a majority of men, older employees, higher proportions of racial minorities, and with jobs not requiring advanced degrees are more likely to have tobacco-users on their payrolls [1].

While these demographic differences in tobacco usage are known, it is not known what the characteristics of worksites are that have successfully implemented tobacco-free policies. Accordingly, this study sought to identify the characteristics of worksites that are most likely to implement such a policy.

## Methods

### Participants

Worksites in Kansas that completed a baseline Phase I WorkWell Kansas (WorkWell KS) Assessment from January 1, 2013 through October 31, 2014 were included in this study. “Participants” were defined at the worksite level; one assessment was completed per worksite. A convenience sampling frame was utilized.

### Procedures

The study utilized a cross-sectional survey design. Worksites that took participated in a WorkWell KS workshop during the study period were eligible for this study. WorkWell KS “Champions,” including KDHE grantees, Chamber of Commerce staff, and local health department staff, were invited by WorkWell KS to recruit worksites across the state to participate, which provided for the largest possible sampling frame of Kansas worksites. The opportunity to participate in WorkWell KS was an opportunity for all workplaces in Kansas, and there was no cost to participate.

Prior to attending their in-person, one and a half day workshop, participating worksites were provided an electronic survey link to complete a baseline survey (see below). Only fully completed surveys from WorkWell KS participants were included in the analysis. Then, at the workshop, participants received reports that were tailored to each participating worksite.

At each worksite, an individual or a team with knowledge of the worksite’s demographics, policies, and practices were asked to complete the assessment. These participants vary in position by worksite, but include worksite executives, human resources directors, and wellness coordinators.

### Instrument

The WorkWell KS survey was developed via literature review of relevant worksite assessments identified by a committee of local health department officials, university physicians, the state department of health and environment, and state civic leaders. This team was originally convened in 2008 as the Kansas Worksite Wellness Advisory Committee.

The instrument is a 120-item instrument organized in three major sections, including: 1) organizational demographics, 2) the worksite wellness foundation, and 3) individual wellness items based on selected health topics and strategic approaches to wellness an individual worksite may have utilized. Ten items (including community size, employer size, occupational classification, community name, ethnic, racial and gender composition of the workforce) were contained in the demographic portion of the instrument. Five health topics, including tobacco, physical activity, healthy foods, well-being, and chronic disease were represented in the questionnaire. A total of 22 items from the survey were designated as tobacco items, four of which were policy strategies to address tobacco in the workplace (Additional file 1).

### Statistical analysis

A dichotomous outcome variable was created to measure the presence of a policy supporting a tobacco-free workplace based on responses to “*Does your worksite have any written policies in place supporting a tobacco-free workplace?”* Demographic variables were presented as frequencies and proportions at univariate level. Bivariate chi-square analyses were conducted to compare the outcome variable by the community sizes of each worksite, number of employees at each worksite, industry classifications, gender composition, ethnic composition, and age distribution at each worksite. A logistic regression analysis was conducted to identify predictors associated with having a tobacco-free policy at the worksite. All variables that were significant at bivariate level were selected as possible predictor candidates for the logistic regression. Interactions between predictors were assessed for significance before a decision was made to include those interactions in the logistic regression model. All statistical analyses were conducted using the SAS software for Windows version 9.3 (Cary, NC). All tests were two-sided, and a *p*-value < 0.05 was considered statistically significant.

## Results

### Worksite and community characteristics

A total of 276 worksites from 29 communities in Kansas were included in the final analysis. The average worksite represented 224 employees, whereas the median employer size was 74 employees (Table 1). Most employers were from rural or ‘non-metropolitan’ communities (74.6%), based on US Census Bureau designations for urban/rural status (Census Bureau, 2015). Thirty-seven percent (37.1%) of worksites reported having fewer than 50 employees, and 37.5% reported having between 50 and 249 employees. The most common type of place of work was in a ‘general office’ (29.5%), followed by ‘hospital or healthcare’ (22.9%), or ‘government’ (17.6%). Most worksites (65.6%) had more females than males, and 88.2% of worksites had a majority of their employees younger than 50 years.

**Table 1:** Community and Worksite Demographics

	Frequency (*N* = 276)	Percent
Population Density		
Rural	206	74.6
Urban	70	25.4
Employer Size		
0-49	102	37.1
50-249	103	37.5
250 or more	70	25.4
*Missing* = 1		
Employer Type		
Agriculture/Manufacturing	31	13.7
General Office	67	29.5
Government	40	17.6
Hospital or Healthcare	52	22.9
Schools or Education	36	15.9
“Other”	1	0.4
*Missing* = 49		
Gender		
> =50% were females	168	65.6
> =50% were males	88	34.4
*Missing*	20	
Age		
1 - 50% of workers who were 50 years and older	202	88.2
51% or more of workers who were 50 years and older	27	11.8
*Missing*	47	
Ethnicity		
Lower than the Kansas Hispanic percentage (11.2%)	196	81.3
Higher than the Kansas Hispanic percentage (11.2%)	45	18.7
*Missing*	35	
Composition of the Workforce		
1-25% manual labor	143	56.75
26-50% manual labor	30	11.9
51-75% manual labor	43	17.06
76-100% manual labor	36	14.29
*Missing*	24	
Tobacco-free Policy		
Has tobacco-free policy	156	56.7
Does not have tobacco-free policy	119	43.3
*Missing*	`	

### Bivariate results

More than half (57%) of all worksites reported they had a policy in place supporting a tobacco-free workplace. First, worksites located in urban counties (76.8%) were significantly more likely than worksites in rural counties (50.0%) to have a policy in place supporting a tobacco-free workplace (*p* < 0.001) (Table 2). Second, worksites with more than 250 employees (74.3%) were significantly more likely to have a tobacco-free policy than worksites with 50 to 249 employees (58.3%) and worksites with fewer than 50 employees (43.1%, *p* < 0.001). Third, worksites in the agriculture/manufacturing sectors (35.5%) were less likely to have a tobacco-free policy than white-collar settings, like those in the hospital/healthcare industrial sector (61.5%) or worksites in the education or school sectors (69.4%, *p* = 0.013). Finally, worksites with predominantly male employees (50%) were significantly less likely to have a tobacco-free policy in place than employers with a majority of female employees (62.9%, *p* = 0.047). Age, ethnicity, and manual labor percentage were not associated with tobacco-free policy in worksite (all three *p*-values > 0.05, Table 2).

**Table 2:** Worksite Tobacco-Free Policies

	No Tobacco-Free Policy (*n* = 119)	Tobacco-Free policy(*n* = 156)	*P-*value
Population Density			0.0001
Rural	103 (50%)	103 (50%)	
Urban	16 (23.2%)	53 (76.8%)	
Employer size			0.0003
0-49	58 (56.9%)	44 (43.1%)	
50-249	43 (41.8%)	60 (58.3%)	
250 or more	18 (25.7%)	52 (74.3%)	
Employer Type*			0.0125
Agriculture/Manufacturing	20 (64.5%)	11 (35.5%)	
General Office	40 (59.7%)	27 (40.3%)	
Government	18 (45%)	22 (55%)	
Hospital or Healthcare	20 (38.5%)	32 (61.5%)	
Schools or Education	11 (30.6%)	25 (69.4%)	
“Other”	1 (100%)	0 (0%)	
*Missing* = 48			
Gender			0.0474
> =50% were females	62 (37.1%)	105 (62.9%)	
> =50% were males	44 (50%)	44 (50%)	
*Missing* = 20			
Age			0.7233
1 - 50% of workers who were 50 years and older	82 (40.6%)	120 (59.4%)	
51% or more of workers who were 50 years and older	10 (37%)	17 (63%)	
*Missing* = 47			
Ethnicity			0.1858
Lower than the Kansas Hispanic percentage (11.2%)	79 (40.3%)	117 (59.7%)	
Higher than the Kansas Hispanic percentage (11.2%)	23 (51.1%)	22 (48.9%)	
Frequency Missing = 35			
Composition of the workforce			0.1099
1-25% manual labor	56 (39.2%)	87 (60.8%)	
26-50% manual labor	14 (46.7%)	16 (53.3%)	
51-75% manual labor			
76-100% manual labor	22 (61.1%)	14 (38.9%)	
Frequency Missing = 24			

*p-value calculation was based on the exclusion of *other* category in the employer type

### Logistic regression results

A multivariable logistic regression was conducted to identify factors associated with worksites having a tobacco-free policy. Possible factors included: population density, employer size, employer type, and gender composition. Worksites in rural areas were significantly less likely to have tobacco-free policies in place than worksites in urban areas (OR = 0.47, 95% confidence interval (CI): 0.23-0.95) (Table 3). Likewise, small employers (with between 1 and 49 employees) were significantly less likely than large worksites (with more than 250 employees) to have tobacco-free policies in place (OR = 0.34, 95% CI 0.16, 0.71). Medium-sized employers (between 50 and 249 employees) were less likely to have a tobacco-free policy in place than large-sized employers (OR = 0.54, 95% CI 0.26, 1.12), though the difference failed to reach statistical significance (Table 2).

**Table 3:** Odds Ratio Estimates with 95% Confidence Interval for Having a Tobacco-Free Policy

	Unadjusted Odds Ratio	Adjusted Odds Ratio
Population Density		
Rural	0.31 (0.16, 0.57)	0.47 (0.23, 0.95)
Urban	Reference	Reference
Employer Size		
1-49	0.27 (0.14, 0.52)	0.34 (0.16, 0.71)
50-249	0.48 (0.25, 0.94)	0.54 (0.26, 1.12)
250 or more	Reference	Reference
Employer Type		
Agriculture/Manufacturing	0.24 (0.09, 0.67)	Not significant in multivariable logistic regression
General Office	0.3 (0.13, 0.7)	
Government	0.54 (0.21, 1.38)	
Hospital or Healthcare	0.7 (0.29, 1.74)	
Schools or Education	Reference	
Gender		
> =50% were females	Reference	Not significant in multivariable logistic regression
> =50% were males	0.59 (0.35, 1)	
Composition of the workforce		
1-25% manual labor	2.44 (1.15, 5.17)	Not significant in multivariable logistic regression
26-50% manual labor	1.8 (0.67, 4.79)	
51-75% manual labor	1.65 (0.67, 4.04)	
76-100% manual labor	Reference	
Age		
1 - 50% of workers who were 50 years and older	0.86 (0.38, 1.97)	Not significant in multivariable logistic regression
51% or more of workers who were 50 years and older	Reference	
Ethnicity		
Lower than the Kansas Hispanic percentage (11.2%)	Reference	Not significant in multivariable logistic regression
Higher than the Kansas Hispanic percentage (11.2%)	0.65 (0.34, 1.24)	

## Discussion

More worksites had tobacco-free policies in place (56.7%) than those that did not (43.3%). This percentage (56.7%) lags behind the percentages in other state-based studies, as it is less than that of the 90% of worksites in Texas with tobacco policies [37], and the 68% of Minnesota worksites with written tobacco policies [38], but there were important differences in how likely a given worksites was to have such a policy at a bivariate and univariate level.

Worksites in urban counties and worksites with more employees were more likely to have tobacco-free policies in place than worksites in rural areas and worksites with fewer employees. These differences are especially important given that smoking is more prevalent in rural areas [39] and that the majority of employees in Kansas’ urban areas worked for large employers with greater than 500 employees [40, 41]. In fact, 27% of adults in rural areas report being current smokers, whereas 18% of adults in large metropolitan statistical areas (MSAs) are current smokers [1].

Large worksites in Kansas were nearly three times as likely as small employers to support tobacco-free policies. This is similar to a pattern observed in a similar statewide study of Minnesota employers, which suggested a 36% increased likelihood for large employers to have written tobacco policies in place more than smaller employers (89% v 65%) [38]. In a largely rural and geographically large state such as Kansas, it is especially important for rural and smaller employers to more fully embrace tobacco-free policies in the workplace, as the majority of worksites in the sample were located in rural, non-metropolitan statistical area communities (76%) and had fewer than 250 employees (76%).

In addition to the differences at the community and worksite level that are perhaps best addressed through policy, it is also important to consider individual-level demographic characteristics in tobacco use that can also be addressed through evidence-based cessation programs (e.g., counseling and medication). A tobacco-free policy, implemented in conjunction with evidence-based smoking cessation programs, can double the likelihood that an employee will stop smoking [42].

The current study adds to individual-level data and risk factors in use of tobacco by examining the worksite and its policies. This focus on population health is a necessary shift, as worksite policies, when implemented with other evidence-based strategies, can influence the employer’s financial health and productivity in addition to improving the physical health of employees, on and off the job.

### Limitations

These are self-reported data; employers completed these surveys in advance of a worksite wellness workshop for which they had been recruited to participate. While WorkWell KS was publically promoted, and participation was actively solicited via WorkWell KS Champions statewide, this sample does not necessarily represent Kansas worksites. Even though the workshop was free, participation required a time commitment from participants, which could have resulted in selection bias. It could be assumed that the entire sample was at least somewhat interested in worksite wellness, although the WorkWell KS Champions were charged with recruiting worksites that were influential in their communities, not necessarily those with an interest in health. Additionally, the social pressure to give the ‘right’ answers may have inflated some results. However, there is no reason to assume any response biases were not evenly distributed across worksites by community size or worksite size; any patterns of differences observed were likely unaffected by any possible response biases. Finally, this study was conducted in a cross-sectional design, so causation is impossible to determine.

## Conclusions

Tobacco policies were common across participating worksites, but important differences exist. Rural and small employers, which are the predominant types of employers in Kansas, were less likely to have tobacco-free policies in place compared to their urban and large counterparts. Future policies and programs that attempt to address tobacco usage in rural areas and among small employers may need to make more comprehensive efforts to reducing tobacco use.

## Additional file

**Additional file 1:**.(PDF 619 kb)
