# Supplementary material for: Tobacco-free policies at worksites in Kansas
Source: BMC Public Health. 2017 Jun 12;17:566. doi: 10.1186/s12889-017-4277-9 (PMC5468950; doi:10.1186/s12889-017-4277-9)
Supplement: Supplementary file 1 — Additional file 1:.(PDF 619 kb) [file 12889_2017_4277_MOESM1_ESM.pdf]

Kansas Worksite Well 2013

Introduction

The Kansas Department of Health and Environment, Bureau of Health Promotion offers this survey as part of the Healthy Kansas Worksite Initiative. Developed by the Bureau of Health Promotion and a committee of experts from various industrial sectors and geographic regions in Kansas, this survey is designed to function as a resource for Kansas employers attempting to enhance the health of their employees. You have been contacted regarding this survey based upon your affiliation with the WorkWell Kansas and you will use your results from this survey as a baseline measure of your worksite wellness efforts in preparation of taking part in a WorkWell Kansas seminar in your city. This survey will take approximately 60 minutes to complete, and your participation is completely voluntary.

Who is to Complete this Survey?

• A human resource professional or the individual tasked with coordinating wellness efforts for the business/organization can answer all of the questions for your business/organization. If you would prefer to obtain a well-rounded viewpoint of the worksite, multiple individuals at the organization might provide input to complete the survey. If so, a PDF copy can be made available to facilitate the process of obtaining input from multiple stakeholders. However, all responses must be submitted online, and only one survey is to be completed per worksite.

How Do You Complete the Survey?

• Before beginning the survey, it is recommended that you **have a copy of your organization's benefit plan (if applicable), mission statement (if applicable), and/or any basic demographic data of your employee population at your disposal to reference**. Having this available will enable you to respond as quickly and accurately as possible.
• You will be asked to provide basic information about yourself (the primary person completing the online survey) and your worksite. After that, the survey is organized in several sections, with the questions in each section assessing your wellness efforts at your worksite in each topic area **over the past 12 months.**
• Questions which require a response will be indicated by an asterisk (*).
• You may exit the survey at any time and return, using the same survey link, to complete your responses at a later date.
• There will be a "percent completed" bar at the top of the survey to help you track your progress.

Privacy and Confidentiality

Your individual responses to the survey will not be shared with anyone outside of the KDHE Bureau of Health Promotion or the University of Kansas School of Medicine-Wichita. This survey will be used as an assessment to provide to worksites. Aggregated data, with no company identifiers, may be used for normative or research purposes, and aggregate results of research studies may be published. If you have any questions regarding this survey, please contact Dr. Elizabeth Ablah at 316-293-2627 (eablah@kumc.edu).

How Will My Organization Benefit From Completing this Survey?

• Your company will receive a report of customized results as well as feedback. Your worksite will also be eligible to receive a wellness plan after survey completion.

If you are prepared to begin, please select **Next**.

Kansas Worksite Well 2013

Preliminary Information

* 1. Please provide the following information about yourself and your business/organization.

Name of business/organization:

Name of primary individual completing survey:

County:

ZIP:

Email address:

Phone number (including area Code):

Kansas Worksite Well 2013

Organizational Demographics

* 2. Not counting temporary or seasonal employees, please estimate how many employees and their dependents currently work for your business/organization?

Approximate number of employees: [ ]

Approximate number of employee dependents (spouses and children): [ ]

* 3. Approximately what percentage of your employees are ...

Full time (as defined by your business/organization)? [ ]

Part time (as defined by your business/organization)? [ ]

* 4. Excluding any security staff, which of the following work shifts does your worksite have? (*select all that apply*)

- [ ] 1st (Approx 8AM - 4PM)
- [ ] 2nd (Approx 4PM - 12AM)
- [ ] 3rd (Approx 12AM - 8AM)

* 5. Please provide the total number of your business/organization's worksites, including all satellite offices (geographically dispersed worksites not managed as a single location).

[ ]

* 6. Approximately what percentage of your employees have primary job duties that would classify their work as ...

manual labor? [ ]

non-manual labor? [ ]

Employee Demographics

* 7. Approximately what percentage of your employees are ...

between 18 - 29 years old?

between 30 - 49 years old?

between 50 - 64 years old?

65 or older?

* 8. Approximately what percentage of your employees are ...

Male?

Female?

* 9. Approximately what percentage of your employees are ...

American Indian or Alaska Native?

Asian?

Black or African American?

Native Hawaiian or Other Pacific Islander?

White?

Other?

* 10. Approximately what percentage of your employees are ...

Hispanic or Latino?

Not Hispanic or Latino?

Wellness Foundations

Building a solid foundation is the key to which successfully developing a comprehensive worksite wellness program is predicated upon. The following items assess which of these foundation steps your worksite or organization has already taken and which area in which your worksite or organization might benefit from 'shoring up the foundation' before moving forward on developing your comprehensive worksite wellness program.

Critical foundational items for any worksite include establishing a wellness committee, using data effectively, securing buy-in from leadership, communicating a consistent wellness message, and developing incentives for participating in wellness acitivities.

* 11. Has your worksite implemented any type of wellness initiatives in the past 12 months?

- ◯ Yes
- ◯ No
- ◯ I Don't Know

12. For approximately how many years has your worksite's wellness program been in operation? (If not applicable, please type N/A)

[ ]

* 13. Does your worksite wellness committee develop a yearly plan with objectives?

- ◯ Yes
- ◯ No
- ◯ I Don't Know
- ◯ Not applicable

* 14. Does your worksite wellness committee celebrate important successes?

- ◯ Yes
- ◯ No
- ◯ I Don't Know
- ◯ Not applicable

Wellness Foundations - Wellness Committees

15. Does your worksite have at least one individual responsible for wellness?

- Yes
- No
- I Don't Know
- Not Applicable

16. Does the individual responsible for worksite wellness have these job duties included in his/her position description?

- Yes
- No
- I Don't Know
- Not Applicable

17. Does your worksite have a wellness committee?

- Yes
- No
- I Don't Know
- Not Applicable

18. Does your worksite's wellness committee have a budget?

- Yes
- No
- I Don't Know
- Not Applicable

19. Does your worksite's wellness committee have representation from all departments and levels of management?

- ○ Yes
- ○ No
- ○ I Don't Know
- ○ Not Applicable

Kansas Worksite Well 2013

Wellness Foundations - Data

* 20. During the previous 12 months, has your worksite provided a health risk appraisal assessment to employees?

- Yes
- No
- I Don't Know

* 21. Have employees been asked to provide feedback on any offered wellness initiatives (e.g., client satisfaction surveys, records from a suggestion box)?

- Yes
- No
- Not applicable
- I Don't Know

* 22. Does your worksite conduct program evaluation of wellness programs/activities (e.g., track participation rates, compare outcomes to pre-determined objectives/goals)?

- Yes
- No
- Not Applicable
- I Don't Know

23. If you have a worksite wellness committee or coordinator, does the committee/coordinator have access to data such as employee medical costs, personnel records, absenteeism, etc.?

- Yes
- No
- I Don't Know
- Not applicable

* 24. Does your worksite measure the return on investment (ROI) for health and wellness efforts?

- Yes
- No
- Not Applicable
- I Don't Know

If 'yes', how is ROI measured by your worksite?

* 25. Has your worksite completed an assessment of employee*wellness needs* (not a health risk assessment) during the previous 12 months?

- Yes
- No
- I Don't Know

Kansas Worksite Well 2013

Wellness Foundations - Communication

26. Does your worksite feature at least annual messages from the CEO supporting wellness (personal address, memo, newsletter article)?

- Yes
- No
- I Don't Know
- Not applicable

27. If your worksite has recently implemented wellness initiatives, did you have a communications plan as part of the initiative?

- Yes
- No
- I Don't Know
- Not applicable

28. If you have a worksite wellness committee or coordinator, does the committee/coordinator regularly report successes and challenges of any wellness initiatives?

- Yes
- No
- I Don't Know
- Not applicable

29. Does your worksite communicate wellness messages to employees using multiple modalities, such as emails, blogs from the CEO, newsletters, department-wide meetings, social media, etc.?

- Yes
- No
- I Don't Know
- Not applicable

Wellness Foundations - Leadership

* 30. Has your worksite established goals for wellness?

- Yes
- No
- I Don't Know

* 31. Does your worksite's organizational mission statement contain references to improving/maintaining employee health?

- Yes
- No
- I Don't Know

32. If your worksite has recently implemented wellness initiatives, did you have executive support?

- Yes
- No
- I Don't Know
- Not Applicable

33. If your worksite has recently implemented wellness initiatives, was executive support evident by presence at kick-off events, in the provision of company resources to support the initiative?

- Yes
- No
- I Don't Know
- Not Applicable

* 34. Does your worksite's executives foster a healthy culture?

- Yes
- No
- I Don't Know
- Not applicable

* 35. Do your worksite's executives share a vision of wellness?

- Yes
- No
- I Don't Know
- Not applicable

* 36. Do your worksite's executives commit resources to wellness?

- Yes
- No
- I Don't Know
- Not applicable

Kansas Worksite Well 2013

Wellness Foundations - Incentives

* 37. Does your worksite or health plan offer employees incentives to participate in health screenings or assessments/appraisals?

- Yes
- No
- I Don't Know

* 38. Does your worksite have budget allocations for incentives tied to worksite wellness initiatives?

- Yes
- No
- I Don't Know

* 39. Does your worksite use a range of incentives to support wellness initiatives to include recognition, merchandise, monetary rewards, time off, etc. ?

- Yes
- No
- I Don't Know
- Not applicable

* 40. Does your wellness committee you have a list of at least 10 incentives of interest to employees?

- Yes
- No
- I Don't Know
- Not applicable

Kansas Worksite Well 2013

Information

* 41. Does your worksite provide employees information (e.g., brochures, videos, posters, pamphlets, newsletters) addressing the benefits of ... ?

	Yes	No	I Don't Know
Physical activity	○	○	○
Nutrition	○	○	○
Tobacco cessation	○	○	○
Good mental health	○	○	○
Chronic Disease management	○	○	○

* 42. Does the worksite sponsor or promote guest speakers or lecturers on ...?

	Yes	No	I Don't Know
Physical activity	○	○	○
Nutrition	○	○	○
Tobacco cessation	○	○	○
Mental health and stress management	○	○	○
Chronic Disease management	○	○	○

* 43. Does your worksite promote or sponsor health fairs or other types of events were screening or educational services are provided to employees for ...

	Screening	Education	I Don't Know
**A.** Cancers recommended for routine screening such as breast, cervical, prostate, or colorectal	☐	☐	☐
**B.** Chronic conditions such as diabetes, high blood pressure, or lipid (cholesterol) disorders	☐	☐	☐
**C.** Mental health issues such as alcohol misuse, anxiety disorders, and depression	☐	☐	☐
**D.** Healthy diet and nutrition	☐	☐	☐
**E.** Physical activity	☐	☐	☐

* 44. Does anyone in the worksite know how to adapt physical activities for employees with mobility disabilities?

- Yes
- No
- I Don't Know

* 45. Does your worksite provide nutritional labeling (e.g., ‘low fat,’ ‘light,’ ‘heart healthy,’ ‘no trans fat’) at the worksite’s cafeteria and onsite food service?

- Yes
- No
- I Don't Know
- Not applicable

* 46. Has your worksite adopted curricula or training to raise awareness of the signs and symptoms of heart attacks and strokes for employees?

- Yes
- No
- I Don't Know

* 47. Does your worksite utilize signage, posters, or brochures to promote chronic disease prevention (e.g., post signs reminding employees to get blood pressure checked, quit smoking, or avoid secondhand smoke) to employees?

- Yes
- No
- I Don't Know
- Not applicable

* 48. During the past 12 months, did your worksite offer employees access to a nationally-recognized training course on:

	Yes	No	I Don't Know
**A.** Automated External Defibrillator (AED) usage?	◯	◯	◯
**B.** Cardiopulmonary Resuscitation (CPR)?	◯	◯	◯

* 49. Did your worksite provide anti-tobacco educational messages to the general employee population beyond the basic signage required by the Kansas Indoor Clean Air Act during the previous 12 months?

- Yes
- No
- I Don't Know

* 50. Has your worksite conducted targeted health promotion campaigns, focusing on key health behaviors and use of preventive health care?

- Yes
- No
- I Don't Know
- Not applicable

* 51. Has your worksite conducted a sustained, focused media and educational campaigns (using multiple modes) to increase consumption of specific healthful foods or reducing consumption of specific less healthful foods or beverages?

- Yes
- No
- I Don't Know
- Not applicable

* 52. Has your worksite offered stress reduction presentations (conflict resolution, managing multiple priorities, personal finance planning)?

- Yes
- No
- I Don't Know
- Not applicable

* 53. Has your worksite conducted a sustained, focused media and educational campaign to promote physical activity?

- Yes
- No
- I Don't Know
- Not applicable

* 54. Has your worksite conducted a sustained, focused media and educational campaign to reduce tobacco use?

- Yes
- No
- I Don't Know
- Not applicable

Program

* 55. Has your worksite offered the evidence-based arthritis program, Walk with Ease?

- Yes
- No
- I Don't Know
- Not applicable

* 56. Has your worksite offered an evidence-based Chronic Disease Self-Management program?

- Yes
- No
- I Don't Know
- Not applicable

* 57. Does your worksite have a program in place to provide free or subsidized lifestyle self-management programs that include tools on ... (can be delivered through vendors, onsite staff, online, health plan, community groups, or other practitioners)?

	Yes	No	I Don't Know
Physical activity			
Nutrition			
Tobacco cessation			
Mental health and stress management			
Chronic Disease management			

* 58. Does your worksite have a program that features a corporate agreement with a health club or gym to offer your employees discounted or subsidized memberships?

- Yes
- No

* 59. If your worksite has stairways, does it have a program in place to promote stair usage in the last 12 months?

◯ Yes

◯ No

◯ I Don't Know

◯ Not applicable

* 60. Does your worksite promote parking farther away to encourage more physical activity?

◯ Yes

◯ No

◯ I Don't Know

◯ Not applicable

* 61. Does your worksite promote walking at work during breaks to encourage more physical activity?

◯ Yes

◯ No

◯ I Don't Know

◯ Not applicable

* 62. Does your worksite have a program in place to faciltate the purchasing of fresh produce or other nutritious food?

◯ Yes

◯ No

◯ I Don't Know

◯ Not applicable

* 63. Has your worksite directly provided or promoted smoking cessation programs/services during the previous 12 months?

◯ Yes

◯ No

◯ I Don't Know

* 64. Does your worksite have a program to support access to tobacco treatment (e.g., nicotine replacement therapy) for those who wish to quit by:

	Yes	No	I Don't Know
**A.** Providing free or subsidized smoking cessation counseling (can be delivered on or off site through vendors, on-site staff, health insurance plans, or other practitioners in a group or individual setting)?	◯	◯	◯
**B.** Referring smokers to a state (e.g., 1-800-QUIT NOW) quit line?	◯	◯	◯
**C.** Referring smokers to other tobacco cessation telephone quit line?	◯	◯	◯

* 65. Does your worksite provide an employee assistance program (EAP)?

◯ Yes

◯ No

◯ I Don't Know

* 66. Has your worksite provided managers with training to reduce workplace stress-related issues during the previous 12 months?

◯ Yes

◯ No

◯ I Don't Know

* 67. Has your worksite provided or promoted programs for stress or related issues during the previous 12 months?

◯ Yes

◯ No

◯ I Don't Know

* 68. Did your worksite sponsor or organize regular social events (e.g., company picnic, holiday party, employee sports teams) during the previous 12 months?

◯ Yes

◯ No

◯ I Don't Know

* 69. Does your worksite have a program to counsel during compony-sponsored activities such as health fairs or pre-employment physicals regarding…

	Yes	No	I Don't Know
**A.** Cancers recommended for routine screening such as breast, cervical, prostate, or colorectal	☐	☐	☐
**B.** Chronic conditions such as diabetes, high blood pressure, or lipid (cholesterol) disorders	☐	☐	☐
**C.** Mental health issues such as alcohol misuse, anxiety disorders, and depression	☐	☐	☐
**D.** Healthy diet and nutrition	☐	☐	☐
**E.** Physical activity	☐	☐	☐

Benefits

70. Does your worksite offer employees health and medical benefits?

- Yes
- No

71. Which statement best represents your worksite's health benefit plan?

- Company purchases coverage from an external agency, such as an insurance company, but does not play a role in the design or administration of health benefits.
- Company operates own health insurance plan. For example, the worksite pays a third party (e.g., an insurance company or health care claims firm) to administer a plan which the worksite has designed for their employees. The employer pays the costs (claims plus administration) out of the company's coffers.

72. On average, what percentage of *employee* health insurance premium is covered by the worksite?

- Less than 60%
- 60-69%
- 70-79%
- 80-89%
- 90-99%
- 100%

73. On average, what percentage of *family* health insurance premium is covered by the worksite?

- Less than 60%
- 60-69%
- 70-79%
- 80-89%
- 90-99%
- 100%

74. Does your worksite's benefit plan include coverage for any *dental* insurance?

- Yes
- No

75. Does your worksite's benefit plan include coverage for any *vision* insurance?

- Yes
- No

* 76. Does your basic benefit package for employees include incentives such as premium reductions for enganging in desireable health behaviors such as ...

	Yes	No	I Don't Know
Maintaining or reaching a certain level of physical activity	○	○	○
Eating a more nutritious diet	○	○	○
Tobacco cessation	○	○	○
Seeking mental health or stress management services (including substance abuse)	○	○	○
Effectively managing chronic conditions such as hypertension or diabetes	○	○	○

* 77. Does your worksite's health insurance benefit include coverage that incentivies being screened for any of the following conditions or health needs ...

	Yes	No	I Don't Know	Not Applicable
**A.** Cancers recommended for routine screening such as breast, cervical, prostate, or colorectal	☐	☐	☐	☐
**B.** Chronic conditions such as diabetes, high blood pressure, or lipid (cholesterol) disorders	☐	☐	☐	☐
**C.** Mental health issues such as alcohol misuse, anxiety disorders, and depression	☐	☐	☐	☐
**D.** Healthy diet and nutrition	☐	☐	☐	☐
**E.** Physical activity	☐	☐	☐	☐

* 78. Does your worksite's benefit plan support access to tobacco treatment for those who wish to quit by providing health insurance coverage that reduces or eliminates employee out-of-pocket costs for FDA-approved:

	Yes	No	I Don't Know
**A.** prescription cessation medications including nicotine replacement (inhaler, nasal spray), bupropion (Zyban) and varenicline (Chantix)?	○	○	○
**B.** over-the-counter nicotine replacement products (e.g., gum, patch, lozenge)?	○	○	○

* 79. Including incentives tied to your company-sponsored insurance company, does your worksite provide any type of benefit for being a non-smoker or quitting smoking?

- Yes
- No
- I Don't Know

If 'Yes", please describe the type of incentive(s) offered.

* 80. Does your worksite provide full coverage for annual influenza vaccinations?

- Yes
- No
- I Don't Know
- Not applicable

* 81. Does your worksite's health plan send reminders to members and network providers about preventive health services?

- Yes
- No
- I Don't Know
- Not applicable

* 82. Does your worksite's health plan require health plans to track delivery of preventive health services and send performance feedback to network providers?

- Yes
- No
- I Don't Know
- Not applicable

* 83. Does your worksite reimburse employees for active commuting (walking/running or biking) to work?

- Yes
- No
- I Don't Know
- Not applicable

* 84. Does your worksite's health plan cover visits with a mental health provider?

- Yes
- No
- I Don't Know
- Not applicable

Policy

* 85. Does your worksite have any written policies in place supporting ...

	Yes	No	I Don't Know
Employee physical activity	◯	◯	◯
Employee access to healthy foods	◯	◯	◯
Tobacco-free workplace	◯	◯	◯
Time and accomodations for mental health and stress management	◯	◯	◯
Chronic disease management	◯	◯	◯

* 86. Does your worksite have a policy providing any type of incentive for engaging in any physical activities?

◯ Yes

◯ No

◯ I Don't Know

87. Does your worksite have a policy providing for flexible work arrangements or break times for employees to engage in physical activity?

◯ Yes

◯ No

* 88. Does your worksite support a flex-time policy that allows employees to adjust their work schedules to accomodate physical activity?

◯ Yes

◯ No

* 89. Does your worksite have a *written* policy making healthy food options (e.g., vegetables, fruits, or low-fat snacks) available during meetings or anytime when food is served?

◯ Yes

◯ No

◯ I Don't Know

* 90. If your worksite has a cafeteria, sells food, or has vending machines:

	Yes	No	I Don't Know	Not Applicable
**A.** Have foods been priced to encourage healthy selections?	◯	◯	◯	◯
**B.** Does your worksite provide labels to identify healthy food choices? (*An example - labels added to food choices by cafeteria staff. It would NOT include information given on the product's own label, such as statements like "light," "low-fat," or "sugar- free.)*	◯	◯	◯	◯

91. Does your worksite's written smoking/tobacco policy include:

	Yes	No	I Don't Know	Not Applicable
**A.** Procedures for ensuring that all employees are made aware of the policy?	◯	◯	◯	◯
**B.** Clear rules around enforcement including penalties for violations?	◯	◯	◯	◯
**C.** Provisions for tobacco/smoking related signage to be posted in the worksite?	◯	◯	◯	◯

* 92. Does your worksite have a *written* sexual harassment policy?

◯ Yes

◯ No

◯ I Don't Know

* 93. Does your worksite have a policy supporting accomodations for mothers to support breast feeding activities at work?

◯ Yes

◯ No

◯ I Don't Know

* 94. Does your worksite have a policy supporting flexible schedule arrangements to facilitate child care needs and schedules for working parents?

◯ Yes

◯ No

◯ I Don't Know

* 95. Does your worksite require and provide sun protection for employees who work outdoors?

- Yes
- No
- I Don't Know
- Not applicable

* 96. Does your worksite subsidize healthful foods and beverages to lower prices?

- Yes
- No
- I Don't Know
- Not applicable

* 97. Does your worksite offer strong nutrition standards for the foods and beverages served at your facility, including vending machines (e.g., policies to reduce transfats, increase fruits and vegetables)?

- Yes
- No
- I Don't Know
- Not applicable

* 98. Does your worksite provide an established time for physical activity during work hours?

- Yes
- No
- I Don't Know
- Not applicable

* 99. Does your worksite require mandatory stretching or stretch breaks?

- Yes
- No
- I Don't Know
- Not applicable

* 100. Does your worksite offer a flex-time policy to employees seeking assistance from a local provider for mental health issues?

- Yes
- No
- I Don't Know
- Not applicable

* 101. Does your worksite have a ban on advertising or promoting tobacco products?

- Yes
- No
- I Don't Know
- Not applicable

* 102. Has your worksite increased parking lot fees to encourage active transportation?

- Yes
- No
- I Don't Know
- Not applicable

Kansas Worksite Well 2013

Environment

* 103. Does your worksite provide any of the following?

	Yes	No
**A.** Maintained Walking Trails	◯	◯
**B.** Bicycle Racks	◯	◯
**C.** Basketball Court	◯	◯
**D.** Outdoor Open Space Designated for Recreation or Exercise	◯	◯
**E.** Shower/Changing Facility	◯	◯
**F.** Indoor Exercise/Fitness Facility On-site	◯	◯

* 104. Does your worksite provide employees with food preparation and storage facilities such as a microwave oven, sink, refrigerator, and/or kitchen?

◯ Yes

◯ No

◯ I Don't Know

* 105. Does your worksite provide vending machines with healthful and moderately priced food selections to engender health eating among employees?

◯ Yes

◯ No

◯ I Don't Know

* 106. Does your worksite offer/promote a farmer's market or community supported agriculture where fresh fruits and vegetables are sold?

◯ Yes

◯ No

◯ I Don't Know

◯ Not applicable

107. Do you provide safe, unflavored, cool drinking water to employees at no cost?

◯ Yes

◯ No

* 108. Does your worksite permit the sale of tobacco products on site (e.g., vending machines, vendors)?

- Yes
- No
- I Don't Know

* 109. Does your worksite have signage in place promoting a smoke-free/tobacco-free workplace?

- Yes
- No
- I Don't Know

* 110. Does your worksite have child care or daycare facilities on-site for use by working parents?

- Yes
- No
- I Don't Know

* 111. Has your worksite designated a breastfeeding room other than a women's restroom?

- Yes
- No
- I Don't Know

* 112. Does your worksite provide an employees-only lounge (non-cafeteria) where employees can relax?

- Yes
- No
- I Don't Know

* 113. If your worksite includes employees who work on-call for extended periods of time, are provisions made to allow these employees an area to rest/sleep when not working?

- Yes
- No
- I Don't Know
- Not applicable

* 114. Does your worksite have accessible blood pressure monitiors and AEDs?

- Yes
- No
- I Don't Know
- Not applicable

* 115. Does your worksite provide on-site influenza vaccinations annually?

- Yes
- No
- I Don't Know
- Not applicable

* 116. Does your worksite's vending machine include prompts, labels, or icons to allow employees to make healthier choices or only provide healthy foods?

- Yes
- No
- I Don't Know
- Not applicable

* 117. Has your worksite improved stairway access and appeal?

- Yes
- No
- I Don't Know
- Not applicable

* 118. Is your worksite's fitness center appealing (e.g., updated, useful equipment)?

- Yes
- No
- I Don't Know
- Not applicable

* 119. Does your worksite enforce tobacco restrictions at work?

- Yes
- No
- I Don't Know
- Not applicable

Worksite Health Promotion

* 120. Considering interest, readiness, need, and momentum, rate your worksite's priorities in addressing each of the following categories (please prioritize each listed item):

	Top priority (within the next year)	Medium priority (within the next 2-3 years)	Low priority (within the next 5 years)
Tobacco control	◯	◯	◯
Healthy foods	◯	◯	◯
Physical activity	◯	◯	◯
Stress management	◯	◯	◯
Chronic disease management	◯	◯	◯
Employee benefits	◯	◯	◯

Kansas Worksite Well 2013

Thank you for completing the survey!

To submit your responses please make sure to click the "Done" button below. Your individual responses to the survey will not be shared with anyone outside of the WorkWell Kansas or the University of Kansas School of Medicine-Wichita.
